# Evaluating the role of *CHEK2* p.(Asp438Tyr) allele in inherited breast cancer predisposition

**DOI:** 10.1007/s10689-023-00327-2

**Published:** 2023-01-19

**Authors:** Timo A. Kumpula, Susanna Koivuluoma, Leila Soikkonen, Sandra Vorimo, Jukka Moilanen, Robert Winqvist, Tuomo Mantere, Outi Kuismin, Katri Pylkäs

**Affiliations:** 1 Laboratory of Cancer Genetics and Tumor Biology, Cancer and Translational Medicine Research Unit, Biocenter Oulu, Oulu and NordLab Oulu, FI-90014 University of Oulu, Oulu, P.O. Box 5000 Finland; 2 Department of Clinical Genetics, Medical Research Center Oulu and PEDEGO Research Unit, Oulu University Hospital, University of Oulu, Oulu, Finland

**Keywords:** *CHEK2*, Breast cancer, Hereditary predisposition, Cohort study, p.(Asp438Tyr) variant

## Abstract

*CHEK2* is a well-established breast cancer susceptibility gene. The most frequent pathogenic *CHEK2* variant is 1100delC, a loss-of-function mutation conferring 2-fold risk for breast cancer. This gene also harbors other rare variants encountered in the clinical gene panels for hereditary cancer. One of these is *CHEK2* c.1312 G > T, p.(Asp438Tyr) in the kinase domain of the protein, but due to its rarity its clinical significance for breast cancer predisposition has remained unclear. Here, we tested the prevalence of *CHEK2* p.(Asp438Tyr) allele showing enrichment in the Northern Finnish population, in a total of 2284 breast cancer patients from this geographical region. Genotyping was performed for DNA samples extracted from peripheral blood using high-resolution melt analysis. Fourteen *CHEK2* p.(Asp438Tyr) carriers were identified (14/2284, 0.6%, P = 0.67): two in the cohort of breast cancer cases with the indication of inherited disease susceptibility (2/281, 0.7%, P = 1.00) and twelve in the breast cancer cohort unselected for the family history of disease and age at disease onset (12/2003, 0.6%, P = 0.66). This frequency did not differ from the frequency in the general population (10/1299, 0.8%). No *CHEK2* p.(Asp438Tyr) homozygotes were identified. Our results indicate that *CHEK2* p.(Asp438Tyr) carriers do not have an increased risk for breast cancer and the classification of the *CHEK2* p.(Asp438Tyr) variant can be changed from the variant of uncertain significance (VUS) to likely benign for breast cancer.

**Supplementary Information:**

The online version contains supplementary material available at 10.1007/s10689-023-00327-2.

## Introduction

Checkpoint Kinase 2 (*CHEK2)* is an important signal transducer in the DNA damage response pathway, inducing cell cycle arrest and apoptosis upon DNA damage [1, 2]. The most predominant pathogenic variant in several populations in this gene is c.1100delC, which accounts for most of the truncating *CHEK2* variants and is associated with breast cancer with 2.3 relative risk [3, 4]. Several other *CHEK2* alleles have been discovered in breast cancer families, but the majority of these are rare, complicating the risk estimations and the interpretations of their clinical significance. Consequently, most of the risk estimates for rare *CHEK2* missense variants are available only for variant groups aggregated according to affected protein domain [5]. One of these rare missense variants is *CHEK2* c.1312G > T (NM_007194.4, rs200050883, Chr22:29091178, GRCh37) deposited in databases several times as being observed in the clinical testing for hereditary cancer (https://www.ncbi.nlm.nih.gov/clinvar/), but with uncertain interpretations of pathogenicity​ (VUS 14 times, likely benign 4 times). It causes p.(Asp438Tyr) substitution in the kinase domain in a position well conserved in vertebrate species, and belongs to American College of Medical Genetics (ACMG) class 3, uncertain significance (PP3, multiple lines of computational evidence support a deleterious effect). At functional level, this alteration has been shown to cause a 70% reduction in the kinase activity on the CHEK2 substrate BRCA1 (Ser988) [6], but controversially has also been reported to act normally in other experimental settings [7, 8].

Although otherwise extremely rare, according to public databases the *CHEK2* p.(Asp438Tyr) allele is enriched in the Finnish population (Finnish minor allele frequency [MAF] 0.00127 versus European MAF 0.0004659) with the highest carrier frequency of 10/1299 (0.8%, MAF 0.0038) in North Ostrobothnia (gnomAD, https://gnomad.broadinstitute.org/ [9]; SISu, http://www.SISuproject.fi/). This geographical enrichment provides an opportunity to test the association of *CHEK2* p.(Asp438Tyr) with breast cancer susceptibility at the population level. For this purpose, here we have tested the prevalence of *CHEK2* p.(Asp438Tyr) in breast cancer patients with the indication of hereditary disease susceptibility and those unselected for the family history of cancer and age at disease onset, all collected from the North Ostrobothnia area.

## Materials and Methods

### Ethical Compliance

This study included informed consent from all participating individuals and was approved by the Ethical Board of the North Ostrobothnia Health Care District.

### Breast Cancer Cohorts

The hereditary cohort (n = 281), collected from the North Ostrobothnia area (Oulu University Hospital), included *BRCA1*/*BRCA2*/*PALB2*/*MCPH1* mutation-negative breast cancer cases with the indication of an inherited predisposition to the disease [10–12]. Cases were selected using the following criteria: (1) index cases from families with three or more breast and/or ovarian cancer cases in first- or second-degree relatives (n = 141), (2) index cases from families with two cases of breast, or breast and ovarian cancer in first- or second-degree relatives, of which at least one with early disease onset (< 35 years), bilateral disease or multiple primary tumors (n = 45), (3) two cases of breast cancer in first- or second-degree relatives (n = 36), and (4) breast cancer cases diagnosed at or below the age of 40 (n = 59). The young breast cancer cases were included based on the assumption that when a woman below the age of 40 years develops breast cancer, a hereditary predisposition can be suspected regardless of the family history [13]. The unselected breast cancer cohort consisted of 2003 consecutive breast cancer cases diagnosed at the Oulu University Hospital during the years 2000–2019 (with a mean age of 58 years at diagnosis) and were unselected for the family history of cancer and age at disease onset. Clinical parameters for these cases were obtained from pathology reports.

### Variant Detection

Genotyping was performed for DNA samples extracted from peripheral blood by using high-resolution melt analysis (CFX96, Bio-Rad, Hercules, CA, USA) with Type-It HRM reagents (Qiagen, Hilden, Germany). Verification of all detected *CHEK2* p.(Asp438Tyr) variants were confirmed with Sanger sequencing (ABI3130xl, Applied Biosystem, Foster City, CA, USA). All identified p.(Asp438Tyr) carriers were genotyped for *CHEK2* c.1100delC (MAF 0.01 in North Ostrobothnia, SISu) with direct sequencing (ABI). The used primers are shown in Online Resource Supplementary Table 1.

### Statistical Analyses

Fisher’s exact test was used to compare the carrier frequencies between cases and controls, and clinical parameters between *CHEK2* p.(Asp438Tyr) carriers and non-carriers (IBM SPSS Statistics 26.0 for Windows, IBM Corp., Armonk, NY). The mean age at diagnosis between carriers and non-carriers in the unselected cohort were compared with Mann–Whitney U test. All P-values were two‐sided and values < 0.05 were considered statistically significant.

## Results

Two cases from the hereditary cohort were identified as *CHEK2* p.(Asp438Tyr) carriers (2/281, 0.7%, P = 1.00, odds ratio [OR] = 0.92, 95% confidence interval [CI] = 0.20–4.21, Table 1), and the presence of other germline *CHEK2* variants in them was ruled out [14]. One carrier was diagnosed with breast cancer at the age of 47 and the other had bilateral disease (at the age of 45 and 48, respectively). In these families, there were four additional breast cancer cases available for testing (family members of Her1 and Her2, respectively, Table 2) and two of them were identified as p.(Asp438Tyr) carriers.

**Table 1:** Frequency of *CHEK2* c.1312 G > T, p.(Asp438Tyr) in the studied breast cancer cases and controls

Cohort	N	WT	%	Mut ^b^	%	OR	95% CI	P ^c^
Hereditary BC	281	279	99.3	2	0.7	0.92	0.20–4.24	1
Unselected BC	2003	1991	99.4	12	0.6	0.78	0.34–1.80	0.66
All BC	2284	2270	99.4	14	0.6	0.80	0.35–1.80	0.67
Controls ^a^	1299	1289	99.2	10	0.8			

*BC* breast cancer, *CI* confidence interval, *Mut* mutation, *OR* odds ratio, *WT* wild-type

^a^ Frequency in the general population in Northern Finland obtained from SISu

^b^ All heterozygous

^c^ Fisher’s exact test

**Table 2:** Family history of the identified heterozygous *CHEK2* c.1312 G > T, p.(Asp438Tyr) carriers

Index ID -Cancers/tumors (age at diagnosis)	Breast/ovarian cancer(s) in 1st and/or 2nd degree relatives (age at diagnosis)	Breast/ovarian cancer(s) in 3rd degree relatives (age at diagnosis)	Other cancers in 1st and/or 2nd relatives (age at diagnosis)
Her1 -Bil Br (45, 48)	Br (29) [-], Br (47) [+], Br (u)	-	Leukemia (65), Lung (u)
Her2 -Br (47)	Br and Basalioma (64) [+], Br (u)	Br (62) [-], Br (u)	Pancreatic (50) [+]
Uns1 -Br (71)	Br (72)	-	-
Uns2 -Br (66)	Bil Br (u)	-	-
Uns3 -Br (62)	Br (u)	-	Prostate and Bone (75)
Uns4 -Br (74)	-	-	Stomach (48), Uterine (49), Brain (7)
Uns5 -Br (75)	-	-	Leukemia (u)
Uns6 -Br (50)	-	-	-
Uns7 -Br (53)	-	-	-
Uns8 -Br (57)	-	-	-
Uns9 -Br (53)	-	-	-
Uns10 -Bil Br (44, 47)	-	-	-
Uns11 -Br (54)	-	-	-
Uns12 -Br (58)	-	-	-

*-* : none reported, *Br* breast, *Bil Br* bilateral breast, *u* unknown disease onset age, *Her* hereditary cohort, *Uns* unselected cohort

All tested cases marked as [+], if positive and [-], if negative for *CHEK2* c.1312 G > T, p.(Asp438Tyr)

In the unselected breast cancer cohort, twelve *CHEK2* p.(Asp438Tyr) carriers were identified (12/2003, 0.6%, P = 0.66, OR = 0.78, 95% CI = 0.34–1.80, Table 1). The mean age at disease onset for the carriers was 60 years (range 44–75 years), which was concordant with the mean of the unselected cohort (58 years, range 28–93 years, P = 0.612). Most of the carrier tumors showed negative or weak Ki-67 proliferation marker staining (10/12, 83.3%, P = 0.02, OR 5.26, CI 1.15–24.06), indicating that the variant does not associate with a higher proliferation rate of the tumor cells. No other significant associations with the clinical parameters of the breast tumors were observed (Online Resource Supplementary Table 2). Three of the *CHEK2* p.(Asp438Tyr) carriers had additional breast cancer cases in their first- and/or second-degree relatives (Uns1-3, Table 2) and three had other cancer types in their family (Uns3–5, Table 2), but no samples from the relatives were available for testing. In family Uns6, two healthy females (age 59 and 75, respectively) were tested as *CHEK2* p.(Asp438Tyr) carriers.

Altogether, the frequency of *CHEK2* p.(Asp438Tyr) in the studied breast cancer cohorts (14/2284, 0.6%, P = 0.67, OR = 0.80, 95% CI = 0.35–1.80, Table 1) did not differ from the population frequency (10/1299, 0.8%) in this geographical region. None of the carriers had the *CHEK2* c.1100delC variant and no homozygous cases were observed in any of the cohorts.

## Discussion

The classification of rare, particularly missense variants in established breast cancer susceptibility genes remains a challenge. The evidence from functional studies may often be controversial and systematic case-control comparisons to assess the pathogenicity are not conclusive if the allele frequency in the general population is ultra-low. For *CHEK2*, several rare missense variants have been reported and generally these have been estimated to confer lower breast cancer risk than protein truncating variants [15]. However, it is possible that rare missense variants in the evolutionarily conserved functional sites cause higher cancer risk [16] and risk estimations for individual alleles are needed. One of the rare variants recurrently encountered in clinical testing (ClinVar) is *CHEK2* p.(Asp438Tyr). As this allele shows an enrichment in the Northern Finnish population, it provides an opportunity to assess its association with breast cancer susceptibility.

In the currently analyzed cohorts, *CHEK2* p.(Asp438Tyr) carriers were identified in 0.7% of the cases with the indication of hereditary predisposition to disease and with similar frequency (0.6%) in the breast cancer cases unselected for family history or age at disease onset. This did not differ from the 0.8% carrier frequency in the healthy population controls. Consequently, similar carrier frequencies in the studied cases and the general population argue against association of *CHEK2* p.(Asp438Tyr) with increased breast cancer risk.

To conclude, the current results indicate that the classification of *CHEK2* p.(Asp438Tyr) variant can be changed from VUS to likely benign for breast cancer. Although in some experimental settings the variant has been shown to decrease the functionality of the CHEK2 protein [6, 16], this does not translate into significantly increased breast cancer risk in the carriers, and it is unlikely to be a significant contributor to breast cancer risk at the population level. This result is particularly important for the genetic counseling units in the clinical diagnostics.

## Electronic supplementary material

Below is the link to the electronic supplementary material.

Supplementary Material 1

## Data Availability

The data to support the findings of this study is available on request from the corresponding author.
