# Supplementary material for: Evaluating the role of CHEK2 p.(Asp438Tyr) allele in inherited breast cancer predisposition
Source: Fam Cancer. 2023 Jan 19;22(3):291–4. doi: 10.1007/s10689-023-00327-2 (PMC10276058; doi:10.1007/s10689-023-00327-2)
Supplement: Supplementary file 1 — Supplementary Material 1 [file 10689_2023_327_MOESM1_ESM.pdf]

Evaluating the role of CHEK2 p.(Asp438Tyr) allele in inherited breast cancer predisposition

Familial Cancer

Timo A. Kumpula[1], Susanna Koivuluoma[1], Leila Soikkonen[2], Sandra Vorimo[1], Jukka Moilanen[2], Robert Winqvist[1], Tuomo Mantere[1], Outi Kuismin[2], Katri Pylkäs[1]

[1] Laboratory of Cancer Genetics and Tumor Biology, Biocenter Oulu, NordLab Oulu, P.O. Box 5000, FI-90014 University of Oulu, Oulu, Finland
[2]Department of Clinical Genetics, Oulu University Hospital,
Medical Research Center Oulu and PEDEGO Research Unit, University of Oulu, Oulu, Finland
Correspondence to Katri Pylkäs: katri.pylkas@oulu.fi

Supplementary Table 1. *CHEK2* primers used in the study

Id	Expected product size (bp)	Start Position (Human GRCh37/hg19)	Sequence (5' - 3')
CHEK2 p.(Asp438Tyr) Forward	162	22:29091122	TGAGACTTCTGCCCAGACTTC
CHEK2 p.(Asp438Tyr) Reverse		22:29091264	CCACTGAGAATGCCACTTGA
CHEK2 c.1100delC Forward	132	22:29091770	AGAACTTCAGGCGCCAAGTA
CHEK2 c.1100delC Reverse		22:29091881	TGGCAAGTTCAACATTATTCC

Supplementary Table 2. Tumor parameters of *CHEK2* p.(Asp438Tyr) carriers compared with the tumors of non-carriers

Category	Mut	%	WT	%	p[a]	OR	95 % CI
**T class**							
1	5	45.5	1143	64.0	0.219	0.47	0.14-1.54
2	5	45.5	566	31.7	(1 vs. 2. 3. 4)		
3	0	0.0	56	3.1			
4	1	9.1	20	1.1			
**M class**							
Neg	12	100	2002	97.8	1	-	-
Pos	0	0	45	2.2			
**Node status**							
Neg	6	50.0	1312	64.4	0.367	0.55	0.18-1.72
Pos	6	50.0	725	35.6			
**ER status**							
Neg	2	16.7	276	14.2	0.684	1.21	0.26-5.54
Pos	10	83.3	1666	85.8			
**PR status**							
Neg	2	16.7	439	22.8	1	0.68	0.15-3.10
Pos	10	83.3	1485	77.2			
**HER2 status**							
Neg	12	100	1588	85.4	0.234	-	-
Pos	0	0	271	14.6			
**Grade**							
1 (well diff.)	5	41.7	371	18.8	0.762	1.58	0.43-5.87
2 (mod. diff.)	4	33.3	921	46.7	(1 and 2 vs. 3)		
3 (poorly diff.)	3	25.0	682	34.5			
**Morphology**							
Ductal	8	66.7	1588	77.9	0.095	2.82	0.84-9.42
Lobular	4	33.3	307	15.1	(Lobular vs. all other)		
Medullary	0	0	10	0.5			
Other	0	0	133	6.5			
**Type**							
LumA	10	83.3	1414	76.4	0.293	2.04	0.44-9.40
LumB	0	0	193	10.4	(Triple negative vs. all other)		
HER2	0	0	78	4.2			
Triple negative	2	16.7	165	8.9			
**KI-67 index**							
0 (negative)	1	8.3	146	7.8	0.020	5.26	1.15-24.06
1 (weak)	9	75.0	765	40.9	(0 and 1 vs. 2 and 3)		
2 (moderate)	1	8.3	502	26.9			
3 (strong)	1	8.3	456	24.4			

CI confidence interval, *ER* estrogen receptor, *LumA* luminalA, *LumB* luminalB, *M* primary metastasis, *Mut* variant carrier, *Neg* negative, *OR* odds ratio, *Pos* positive, *PR* progesterone receptor, *T* tumor size, *WT* wild-type.

[a] Fisher's exact test.
